# Gastric Neuroendocrine Carcinoma Presenting as Ascites in a Young Female

**DOI:** 10.7759/cureus.64964

**Published:** 2024-07-19

**Authors:** Yogesh Bade, Prashant Gopal, Amol S Dahale, Abhijeet Karad

**Affiliations:** 1 Medical Gastroenterology, Dr. D. Y. Patil Medical College, Hospital and Research Centre, Dr. D. Y. Patil Vidyapeeth (Deemed to be University), Pune, IND

**Keywords:** endoscopic ultrasound, ascites, gastric, carcinoma, neuroendocrine tumor

## Abstract

Although neuroendocrine tumors (NETs) can occur in any organ, the majority of them occur in the gastrointestinal (GI) tract. We present the case of a 27-year-old female who presented with ascites. She underwent an ascitic fluid analysis, an esophagogastroduodenoscopy (EGDscopy) with biopsies, and a positron emission tomography (PET) scan, all of which culminated in a diagnosis of a poorly differentiated gastric NET (small cell type) with peritoneal metastasis. She was treated with cisplatin and etoposide. Depending on the differentiation and grade, NETs can manifest in a variety of ways. Definitive diagnosis requires histopathological examination and immunostaining. For smaller well-differentiated NETs, management is either endoscopic or surgical resection. For neuroendocrine carcinomas with metastasis, chemotherapy and symptomatic management are advised. This case report highlights the rare presentation of a neuroendocrine carcinoma as well as discusses its diagnostic approach and possible treatment options.

## Introduction

Neuroendocrine tumors (NETs) are rare neoplasms arising from enterochromaffin cells. NETs can involve various organs, including the lungs, gallbladder, skin, pancreas, thyroid, testes, ovaries, and gastrointestinal (GI) tract. Around 55% of NETs occur in the GI tract, and gastric NETs (GNETs) account for 7-8% of all NETs. Overall, GNETs account for 0.3% of all gastric tumors [1].

As per the World Health Organization (WHO) criteria, GNETs are classified into distinct types based on their differentiation, mitotic index, and/or Ki-67 index or MiB-1 index. Type I GNETs account for 70-80%, are small (1-2 cm), and consist of multiple, well-differentiated polypoidal lesions with a MiB-1 proliferation index of less than 3% (low grade). Type II GNETs account for 5-10%, are small (1-2 cm) and multiple, and consist of well-differentiated polypoidal lesions with a MiB-1 proliferation index of 3-20% (intermediate grade). Type III GNETs, which account for 10-20%, are usually single, large, well-differentiated tumors (>2 cm) with a MiB-1 index of more than 20% (high grade). Neuroendocrine carcinomas (NECs) include small cell types, large cell types, and mixed types. They are rare, large, poorly differentiated tumors with a MiB-1 index of more than 20%. The risk of metastasis is 5-10% with type I GNETs, 10-20% with type II GNETs, more than 50% with type III GNETs, and 100% with NECs [2].

## Case presentation

A 27-year-old female with no co-morbidities presented with complaints of painless distention of the abdomen for one month. She had no history of fever, vomiting, jaundice, or decreased urine output. Examination revealed a distended abdomen with diffuse dullness on percussion but no tenderness, palpable lump, or local rise in temperature.

Routine laboratory investigations were unrewarding (Table 1). Ascitic fluid analysis revealed a low serum-ascites albumin gradient (SAAG) of 0.9, a high protein level of 3.2 g/dL, and a leukocyte count of 700/mm^3^ (80% lymphocytes). Adenosine deaminase (ADA) levels were normal (16 U/L), and fluid analysis was negative for malignant cytology.

**Table 1:** Laboratory investigations ALT: alanine transaminase; AST: aspartate transaminase; PT-INR: prothrombin time-international normalized ratio; HbA1c: glycated hemoglobin; CEA: carcinoembryonic antigen; CA-125: cancer antigen 125

Investigation	Observed value	Reference range
Hemoglobin	13.7 g/dL	12.1-15.1 g/dL
Total leukocyte count	8470 cells/μL	4000-11000 cells/μL
Platelets	178000/μL	150000-450000/μL
Total bilirubin	0.9 mg/dL	0.1-1.2 mg/dL
Conjugated bilirubin	0.3 mg/dL	≤0.3 mg/dL
ALT	23 U/L	4-36 U/L
AST	28 U/L	8-33 U/L
Alkaline phosphatase	138 IU/L	44-147 IU/L
Sodium	136 mEq/L	135-145 mEq/L
Potassium	4.8 mEq/L	3.6-5.0 mEq/L
C-reactive protein	1.4 mg/dL	≤5 mg/dL
PT-INR	1.1	≤1.1
Erythrocyte sedimentation rate	10 mm/hr	≤20 mm/hr
Procalcitonin	0.02 ng/mL	≤0.05 ng/mL
HbA1c	5.3%	≤6%
CEA	1.59 ng/mL	0-2.9 ng/mL
Serum albumin	3.6 g/dL	3.5-5.5 g/dL
CA-125	15 IU/L	0-35 IU/mL
Urea	16 mg/dL	5-20 mg/dL
Creatinine	0.6 mg/dL	0.6-1.1 mg/dL

**Figure 1:**
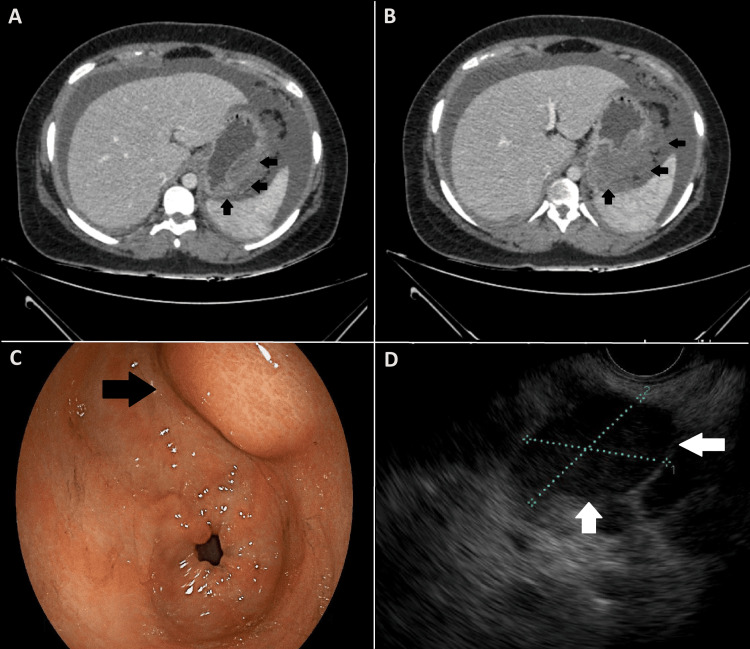
CT, EGDscopy, and EUS images of the NET A and B: A contrast-enhanced CT of the abdomen showing a large ill-defined heterogeneous hypodense mass lesion along the greater curvature of the stomach. C: EGDscopy showing a submucosal lesion along the greater curvature of the stomach. D: An EUS image showing a hypoechoic submucosal lesion. CT: computed tomography; EGDscopy: esophagogastroduodenoscopy; EUS: endoscopic ultrasound; NET: neuroendocrine tumor

A contrast-enhanced computed tomography (CT) of the abdomen revealed a large, ill-defined heterogeneous hypodense mass lesion (8 cm × 7 cm) seen along the greater curvature of the stomach, with a large exophytic component showing heterogeneous post-contrast enhancement likely representing a neoplastic mass (Figure 1A, 1B). Multiple enhancing peritoneal deposits were also noted along the omental surfaces of large bowel loops and diaphragmatic surfaces, representing peritoneal metastases.

A DOTATATE-positron emission tomography (PET) was done, which showed no uptake. A fludeoxyglucose F18 (FDG) PET was done, which showed an FDG-avid mass in the body of the stomach, FDG-avid pelvic, abdominal, mediastinal, and right supraclavicular lymph nodes, and FDG-avid omental, peritoneal, and mesenteric deposits.

Esophagogastroduodenoscopy (EGDscopy) revealed a submucosal lesion along the greater curvature of the stomach (Figure 1C). The mucosa underlying the lesion was normal. An endoscopic ultrasound (EUS)-guided biopsy was taken (Figure 1D).

**Figure 2:**
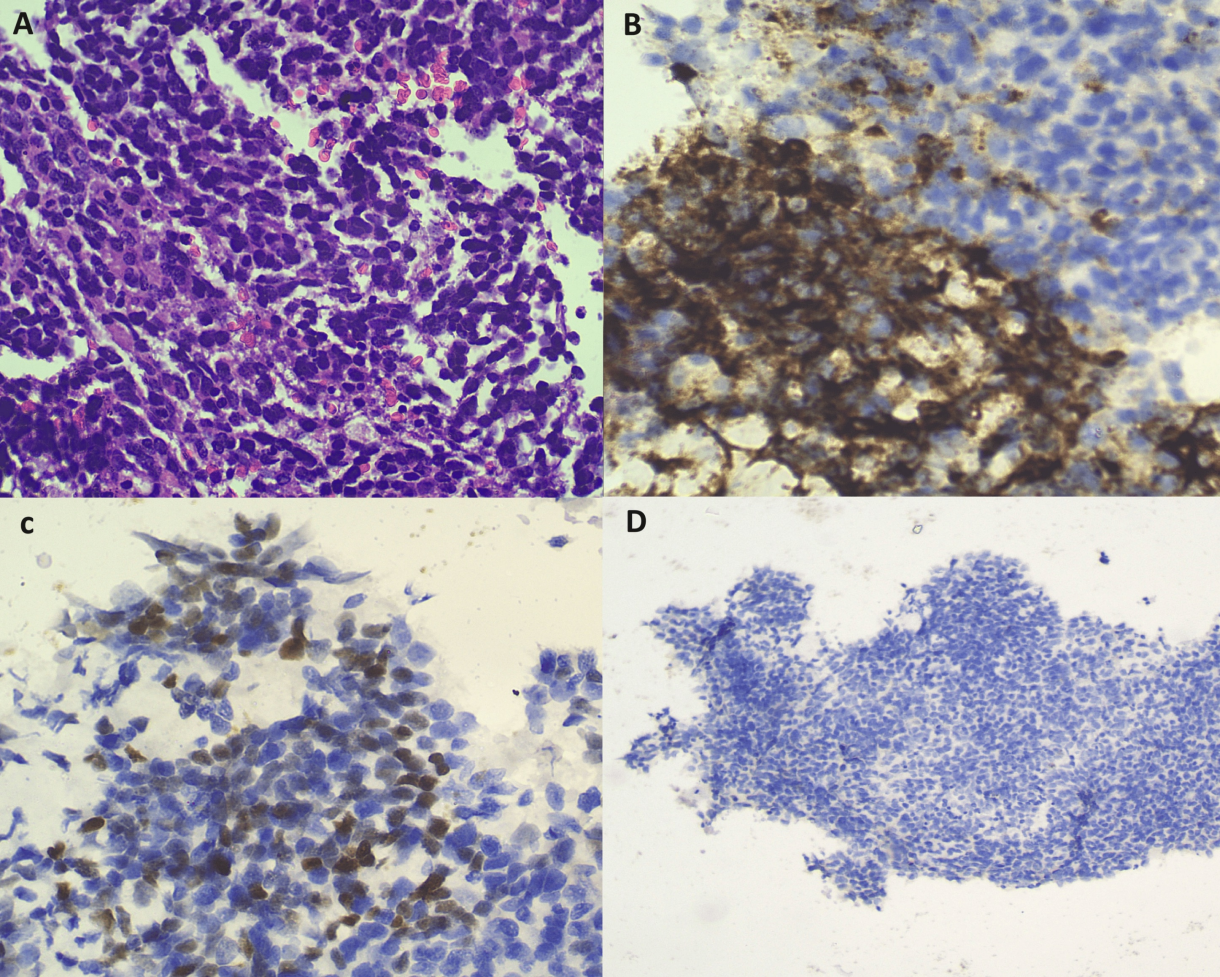
HPE and immunostaining images of the NET cells A: HPE with hematoxylin and eosin staining showing small round neoplastic cells. B: HPE with immunostaining showing positivity for synaptophysin. C: HPE with immunostaining showing focal positivity for EMA. D: HPE with immunostaining negative for chromogranin A. HPE: histopathological examination; NET: neuroendocrine tumor

Histopathological examination (HPE) revealed small round neoplastic cells (Figure 2A) positive for synaptophysin (Figure 2B), EMA (Figure 2C), and CD56 and negative for cytokeratin and chromogranin A (Figure 2D). MiB-1 proliferative index was 50-60% with a mitotic index of >20/mm^2^.

The patient was diagnosed with a poorly differentiated GNETs (small cell type) with peritoneal metastasis. She had recurrent ascites and required large-volume paracentesis (approximately 4 litres) twice a week. Since the tumor was unresectable, the patient was started on cisplatin and etoposide. She had received three cycles of chemotherapy. The ascites abated, and the patient remained asymptomatic. However, there was no reduction in the size of the tumor on imaging. Unfortunately, following the third cycle of chemotherapy, the patient was lost to follow-up.

## Discussion

Diagnosis of NETs is usually done by performing an EGDscopy with HPE of biopsy specimens. Grade I NETs usually have a mitotic rate of <2/mm^2^, grade II 2-20/mm^2^, and grade III >20/mm^2^. Grade I and II NETs usually stain positive for chromogranin A and synaptophysin on immunohistochemical staining. The mitotic rate of NECs is >20/mm^2^, and they usually stain positive for synaptophysin but may be negative for chromogranin A. EUS may be done to look for the depth of the tumor and local lymph node involvement. FDG-PET may not show uptake as NETs are slow-growing tumors. DOTATATE-PET is the preferred imaging modality in patients with suspected NETs. However, in our patient, the DOTATATE-PET showed no uptake, and the lesion was visualized on the FDG-PET scan.

Type I GNETs usually occur in the background of chronic atrophic gastritis and are associated with syndromes like autoimmune polyglandular syndrome. Serum gastrin levels are usually elevated, and the underlying mucosa is usually atrophic. Type II GNETs are associated with Zollinger-Ellison syndrome and multiple endocrine neoplasm-1 (MEN-1) syndrome. Serum gastrin levels are usually elevated, and the underlying mucosa is usually hypertrophic. Type III GNETs are usually sporadic. The serum gastrin levels and the underlying mucosa are usually normal [3].

Patients with type I GNETs can have vitamin B12 deficiency with or without macrocytic anemia due to low levels of intrinsic factor. Antiparietal cell antibodies are positive in 80% of cases. In patients with type I GNETs, smaller lesions (<0.5 cm) are managed conservatively, and larger tumors may be managed endoscopically or surgically. Endoscopic treatment options include polypectomy, endoscopic mucosal resection (EMR), and endoscopic submucosal dissection (ESD). The management of type I and type II GNETs is similar, but the gastrinomas in type II GNETs are resected. Gastrectomy is done for submucosa tumors with or without lymph node involvement and for patients with a positive margin in the resection sample [3].

The management of type III GNETs is similar to that of gastric adenocarcinomas. Total or subtotal gastrectomy along with lymphadenectomy is preferred. If the resected specimen shows poor differentiation on HPE, adjuvant chemotherapy with cisplatin or etoposide is advised. For unresectable NECs, chemotherapy is the only treatment modality.

Folinic acid, fluorouracil, and oxaliplatin, or irinotecan, are second-line treatment options. Other treatment options include peptide receptor radioligand therapy and targeted therapy, including radiofrequency ablation, bland embolization, and chemoembolization. The role of surgery in advanced resectable disease is debated, with a few studies demonstrating survival advantage. However, there was no benefit of surgery in patients with NECs with metastasis [4].

Although response rates are low, somatostatin analogues like octreotide or lanreotide are initiated, especially in the presence of carcinoid syndrome, to reduce the symptoms and long-term complications of uncontrolled carcinoid syndrome. The prognosis of patients with NECs is poor, with an annual mortality rate of up to 57% [4].

## Conclusions

Gastric NEC presenting as ascites in a young female is a rare finding. The incidence of NETs has increased in recent years. Most NETs are slow-growing, while some tumors may show rapid growth. Type I and II NETs have a better prognosis compared to type III NETs and NECs. Management depends on the grading and staging of the tumor. Treatment options include endoscopic and surgical resection and chemotherapy for unresectable tumors.
